# The Role of Myocardial Perfusion Gated SPECT Study in Women with Coronary Artery Disease: A Correlative Study

**DOI:** 10.4274/Mirt.359

**Published:** 2012-08-01

**Authors:** Erdal Nihat Akalın, Olga Yaylalı, Fatma Suna Kıraç, Doğangün Yüksel, Mustafa Kılıç

**Affiliations:** 1 Denizli State Hospital, Department of Nuclear Medicine, Denizli, Turkey; 2 Pamukkale University Medical Faculty, Department of Nuclear Medicine, Denizli, Turkey; 3 Pamukkale University Medical Faculty, Department of Cardiology, Denizli, Turkey

**Keywords:** Atherosclerosis, myocardial perfusion imaging, metabolic syndrome, coronary artery disease

## Abstract

**Objective:** We aimed to evaluate the role of gated myocardial perfusion SPECT (MPS) and to investigate whether only the invasive coronary angiography (CAG) is sufficient in the diagnosis of the coronary artery disease (CAD) in women.

**Material and Methods:** Sixty-four women (62±10 years) with known CAD were included in this study. They had echocardiography (ECHO), stress/rest gated MPS and invasive CAG. Coronary stenosis as of > 50 % in invasive CAG was accepted as significant. Gated MPS data were compared with invasive CAG and ECHO.

**Results:** Invasive CAG results were abnormal in 34 patients, and normal in 30 cases. Myocardial ischemia was detected by gated MPS in 22/ 30 cases with normal invasive CAG, 6 had mild coronary stenosis in major coronary arteries ranging from 30% to 50% in invasive CAG. 16/ 22 women were diagnosed as metabolic syndrome according to MetSend Diagnostic Criteria and only 8 of 30 patients with normal invasive CAG had false positive MPS data on the reevaluation by a nuclear cardiologist.

**Conclusion:** We think that invasive coronary angiography method is not sufficient alone in the diagnosis of CAD in women. Gated MPS study is recommended to achieve the final decision for myocardial ischemia in the cases with CAD and raw data must always be evaluated to avoid attenuation artifacts.

**Conflict of interest:**None declared.

## INTRODUCTION

The incidence of coronary artery disease (CAD) in women increases in the postmenopausal period and becomes equal with men at 60 years of age. It is known that CAD symptoms in women are frequently confused with non-cardiac symptoms and most of the cases are misdiagnosed in emergency units. In western population, two thirds of women who die suddenly from coronary heart disease have no previous history of CAD (1,2,3,4,5). Although the risk factors for CAD are similar in both genders, the risk ratio increases by three-fold for women with systemic diseases such as diabetes mellitus, high LDL cholesterol and obesity. Due to these factors, metabolic syndrome tends to increase with age in women. Additionally, a history of smoking and the use of oral contraceptives are important risk factors for CAD (1,6). In recent years, particularly younger female patients, experienced better improvements than men in hospital mortality after MI. The authors stated that this improvement was largely related to temporal changes in risk profiles (7,8).

Invasive coronary angiography (CAG) is a widely performed procedure in the diagnosis of CAD because treatment is also possible in the same session if a severe occlusion is detected (2,9). However, invasive CAG in the diagnosis of CAD does have a limited importance in female population because of smaller heart size and much thinner epicardial vessels than men (1,10). Therefore, the definition of the severity of luminal obstruction in the thin vessels are too difficult and tricky. Additionally, defining the degree of stenosis in invasive CAG is dependent on operator experience and does not give information about myocardial perfusion. As previously reported, invasive CAG is not adequate to differentiate metabolic syndrome or microvascular disorders yet (11,12,13). To determine regional myocardial perfusion abnormalities and help diagnose metabolic syndrome, MPS study is a valuable method but soft tissue attenuations on scintigraphic images are major limitations. Breast tissue frequently results in ischemic findings especially at anterior and anteroseptal segment in women and generally accepted as false positive regarding to angiography reports (14,15). In these cases, adding gated imaging to MPS study increases the diagnostic accuracy (4,15,16,17,18). On the gated images, left ventricular (LV) wall motion abnormality and decreased systolic thickening at the segment having reduced perfusion show CAD (9,16).

In this study, we aimed to evaluate the role of gated MPS study in the diagnosis of CAD in female patients with myocard perfusion abnormality and second goal is to investigate if the invasive coronary angiography was sufficient alone particularly when the patients have metabolic syndrome. 

## MATERIALS AND METHODS

Sixty-four women who were admitted to our department in the last 3 years with typical angina pectoris, abnormal exercise ECG findings or the inability to perform the treadmill exercise test were enrolled in this retrospective study. All subjects had myocardial ischemia and/or infarction in any left ventricular myocardial segment and underwent invasive CAG within four weeks. Left ventricular systolic function (wall motion and systolic thickening) had also been assessed by 2D Doppler echocardiography (ECHO). All MPS images and gated studies were reevaluated by a nuclear cardiologist. 

Patients’ demographic data are summarized in Table 1. The mean age was 62±10 years. In daily practice, all patients are informed about the procedures and radiopharmaceuticals that will be used, and informed consent form is signed by the patients or their relatives. 

**Gated MPS**

All subjects were instructed to discontinue their medications, such as long-acting nitrates, calcium channel blockers and beta blockers, at least 48 hours before the MPS study according to nuclear cardiology procedure (19,20). At least 12 hours fasting period was requested prior to the test. Stress (treadmill or pharmacological) and rest images were obtained at 45 to 60 mins after radiopharmaceutical injection (99mTc-MIBI or 99mTc-tetrofosmin) according to MPS acquisition guidelines for two day stress/rest myocardial perfusion gated SPECT studies (19,20).

Gated MPS images were obtained with a CamStar AC/T gamma camera (GE-Milwaukee, WI, USA) equipped with LEGP collimator. The raw data was cinematically evaluated for attenuation and motion artifacts, after that, reconstructed stress and rest slices were simultaneously interpreted. Finally, left ventricular regional wall motion and myocardial systolic thickening were evaluated on stress gated images. If there is wall motion abnormality and/or decreased systolic thickening in the myocardial segment having reduced perfusion, this was accepted as true ischemia and if otherwise, not. Motion correction was not needed in any of the cases.

**Invasive CAG Protocol**

Coronary angiography was performed through the femoral artery using the standard Judkins technique in the catheter laboratory of Pamukkale University Medical Faculty, Dept. of Cardiology. Vessel diameter, minimal lumen diameter and the percentage of luminal stenosis were measured using an automated analytic system. Cine-images were evaluated by an experienced cardiologist and lesions over 50% were considered serious coronary stenosis. 

**Doppler ECHO**

Standard and pulsed Doppler tissue ECHO examinations were performed in all cases using a Vivid 7 ultrasound machine (GE Vingmed, Milwaukee, WI, USA) with a 2.5-MHz phased array probe). Left ventricular wall motion and myocardial thickenning were visually evaluated by more than one cardiologist.

## RESULTS

According to invasive CAG results, stenosis of >50% in any vessel (single or multiple) was observed in 34 of 64 women with ischemic gated MPS. In this group, there were a single vessel coronary artery lesion in 18 (53%); 2-vessel disease in 11 (32%), and 3-vessel disease in 5 (15%) patients. Thirty patients were accepted as normal through coronary angiographic evaluation (Table 2). 

Myocardial ischemia was detected on gated MPS images in 22 of 30 cases with normal invasive CAG report. Six of the 22 cases had mild coronary artery stenosis ranging from 30% to 50% in invasive CAG, and the MPS results were matched with invasive CAG findings. Unsignificant stenoses were observed in the LAD in 4 cases and in the RCA in 2 cases. These 6 cases had at least two of 4 risk factors (hypertension, hyperlipidemia, diabetes mellitus, obesity, menopause) and also family history. In these cases, decreased myocardial perfusion was accepted as true ischemia because no soft tissue attenuation artifact was detected in unprocessed data, and ischemic segments were compatible with the localization of mild stenotic lesions on invasive CAG. 

**Figure 1:**
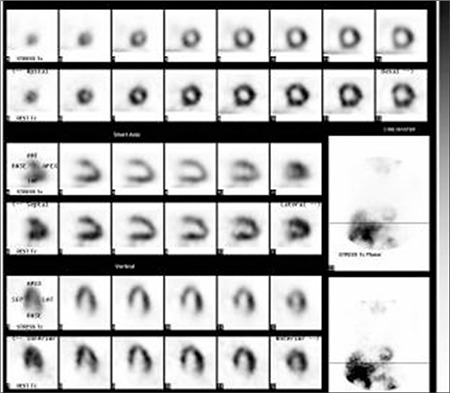
99mTc-MIBI gated MPS images belonging to a case with metabolic syndrome are presented here. A 52 year old female patient had stable angina pectoris, hypertension, diabetes mellitus and positive treadmill exercise test. On two day gated MPS study, anterior and anteroseptal ischemia were determined. In unprocessed data, none breast tissue artifact was detected. Coronary arteries were reported as totally normal by angiography

**Figure 2:**
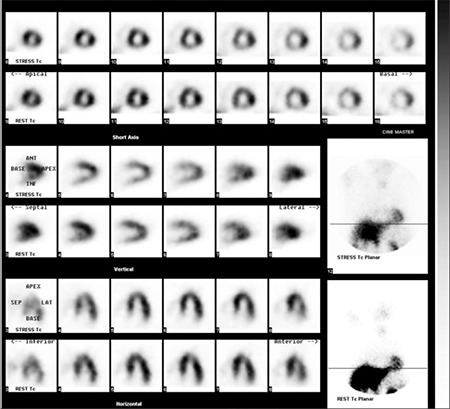
A case for false positive gated MPS study. Normal treadmill exercise test was obtained in a 51 year old female with unstable angina pectoris, hypertension and obesity. Apical and anterior wall perfusion defects were detected in 99mTc-MIBI gated MPS study although invasive coronary angiography results were normal. However, breastattenuation artifact on both stress and rest raw data were observed. Therefore, MPS result was accepted as false positive

Remaining 16/22 cases who had abnormal myocardial perfusion and function but had normal invasive CAG (Figure 1) were defined as endothelial dysfunction secondary to metabolic syndrome. Metabolic syndrome is determined according to the MetSend diagnostic criteria based on having at least three of the following ones: 1) BMI ≥30 kg/m^2^, 2) Diabetes mellitus which is defined being on the usage of antidiabetic medication or having fasting glucose of ≥126 mg/dl, 3) hypertension, 4) hyperlipidemia (fasting triglycerides ≥150 mg/dl and HDL <50 mg/dl) and 5) hormonal changes (menopause) (21,22). None of them had attenuation artifacts on MPS images. Eight of them (50%) were obese; 10 (60%) had hyperlipidemia; 7 (44%) had diabetes mellitus (DM), 12 (75%) had hypertension and 14 (88%) were in menopause. Reduced myocardial perfusion was observed in 8/30 patients (5 in anterior wall and 3 in septum) who had completely normal invasive CAG. In these 8 cases breast attenuation effect was identified on raw data and gated analyses were completely normal at the second evaluation by nuclear cardiologist. Therefore, they were accepted as false positive cases (Figure 2). Looking at body mass index (BMI), obesity was detected in 3 of them. 

Regarding LV wall motion, gated MPS results were unmatched with ECHO findings in 9 of 34 cases having greater than 50% of coronary stenosis, and in 22 of 30 cases without any significant stenotic lesion on invasive CAG. All of the patients had normal ECHO findings in unstenotic group. In stenotic group, ECHO results were normal while gated MPS findings were abnormal in 9/34 cases. Matched gated MPS and ECHO findings were found for remained cases in both groups (Table 2). 

Normal myocardial systolic thickening was detected in all cases which had normal wall motion on both gated MPS study and ECHO. Otherwise, cases with abnormal wall motion on ECHO and gated study showed decreased systolic thickening.

## DISCUSSION

Macrovascular and/or microvascular pathologies cause myocardial perfusion abnormalities which result in LV dysfunction related with the extension and severity of stenotic lesions. Impairment in the LV blood supply and functions can be evaluated by several methods. Treadmill exercise test is the most widely used as a screening test for CAD, but its sensitivity and specificity are low and LV functions can not be evaluated (23). Second preferred test is ECHO because it is an easily applicable, mobile bedside test and there is no radiation exposure risk. Dobutamine ECHO also evaluates biventricular wall motions and systolic thickening besides cardiac volumes under stress (24,25). But, it has limited value in the assessment of myocardial perfusion abnormalities even some microbubble materials are being used intravenously during test. Recently, tissue Doppler technique has been developed to objectively quantify global and regional myocardial systolic and diastolic functions altered by changes in the myocardial blood flow during baseline and stress. But, the confounding effects of cardiac translational motion and passive pathological tethering effect are important limitations which are recently removed with the assessment of strain and strain rate (26). Although these non-invasive tests are available in the most cardiac centers, coronary angiography is still the most preferred test in the diagnosis of atherosclerotic heart disease. Angiography gives detailed anatomic information about atheroma plaques where they localized and what the occlusion severity and extension are. Generally, invasive CAG performed in women may not detect atherosclerotic lesions because atheroma plaques tend to be more diffuse around the arterial wall throughout the vessel and also the coronary arteries are smaller in females. Therefore, definition of coronary lesions are much more difficult even if a severe atheroma plaque is present (3,10). Another risk in women is that non-occlusive atheroma plaques may cause total occlusion on stress and sudden cardiac death (7,27). That’s why, women are accepted as high-risk group in terms of CAD and sudden cardiac death (3,5) and assessment of coronary blood supply to LV myocardium becomes much important in this group. While myocardial perfusion can not be directly evaluated by ECHO or invasive CAG, nuclear cardiac imaging tests provide simultaneous assessment of myocardial perfusion and LV function (24,27). So, defining true myocardial ischemia on the basis of transient regional wall motion abnormalities related to ischemic LV dysfunction is possible (28,29). The good agreement between stress ECHO and gated MPS studies has been reported regarding segmental wall motion analysis (25). 

In this present study, 25 cases of 56 (all had coronary stenosis greater than 50% on invasive CAG images) showed abnormal wall motion on both stress gated MPS and rest ECHO studies. In addition, 9 cases of 34 in abnormal invasive CAG group with abnormal wall motion on gated MPS had normal wall motion on rest ECHO. We thought that this difference might have arised from the gated MPS results which were obtained under stress however the ECHO results were obtained in the rest (baseline) position. Baseline transthorasic ECHO can diagnose real normal cases but mild left ventricular dysfunction may not be truly determined. It was presented in a comparative study with MPS, that the accuracy of myocardial ECHO results were improved during adenosine stress (30). The coronary flow reserve was altered in patients who underwent gated MPS with dipyridamole exercise test and due to this alteration myocardial ischemia was observed by gated MPS. These ischemic findings and the related wall motion abnormalities may not be observed by invasive CAG and also baseline ECHO analysis (30,31). Additionally, test results may be changed related with the patent’s cardiac conditions at the time of tests (time difference is 1 to 4 weeks). We think that gated MPS results are true because they had significant coronary artery lesions and that this discrepancy may be related with the axis of the heart, cardiac translational motion, passive pathological tethering effect on transthorasic ECHO. It may also be related to the interobserver variations due to operator experience causing different results in ECHO. It is known that these are the most important limitations of transthorasic ECHO method (26,28,30). 

Among our study population, 6 patients had ischemic findings on gated MPS accompanied with less than 50% stenosis on invasive CAG. Coronary lesion was detected in LAD in 4 patients and RCA in 2 cases. Similar to our results, Sanlı et al. (32) and Adamu et al. (10) reported that myocardial perfusion defects on MPS study were related with true ischemia localising on where insignificant stenosis was detected by invasive CAG, and stated that, stress MPS has incremental predictive value for future events in patients without significant CAD. Gated MPS is a useful test to determine especially metabolic syndrome and/or microvascular disease in women who are admitted with atypical cardiac symptoms (10,12,32). Gated study provides that myocardial perfusion defects and functional abnormalities can be simultaneuosly evaluated and attenuation artifacts can be differentiated from real perfusion abnormality. Breast tissue is the most common organ causing artifact in MPS images of women (16,33). Similarly with previous reports, breast attenuation was observed in 8 cases with ischemia at the anterior and septal walls in our study. These cases were accepted as false ischemia, only 3 of them were obese. 

Other important disorder is metabolic syndrome in women. In our presented study, metabolic syndrome was diagnosed in 16 of the 30 cases based on the MetSend diagnostic criteria (21,22). Most of them were in the postmenopausal period and hypertensive (88% and 75%, respectively). Almost a half of them were obese, diabetic and hyperlipidemic. These cases were asymptomatic and had no significant artery lesions on invasive CAG. But, all of them had abnormal gated MPS findings. Our findings are matched with previously published reports (1,2,3,4,5,6).

Finally, we suggest that female population should be carefully evaluated for diagnosing CAD. Coronary angiography without gated MPS is not efficient alone in the diagnosis of ischemic heart disease in women. Also, baseline ECHO may not detect transient LV dysfunction resulting from mild to severe ischemia under stress and operator dependency is the major limitation. The gated MPS study will be useful in this risky group to define a reduced myocardial perfusion, and raw data must be evaluated to avoid attenuation artifacts. In the presence of ischemic findings on gated MPS images without severe stenotic lesion on invasive CAG images, CAD is suggested for diagnosis. 

Study limitations: Our study population is not large in this retrospective study. Total 97 females with invasive coronary angiography who are admitted for myocardial perfusion study were evaluated. But, 33 cases were excluded from our study because MPS scan was performed without gated procedure. The gated MPS scan was performed for the remaining 64 patients. Second limitation is that we did not find sensitivity, spesificity, PPV and NPV values because study population is not sufficient for reliability. 

## Figures and Tables

**Table 1:**
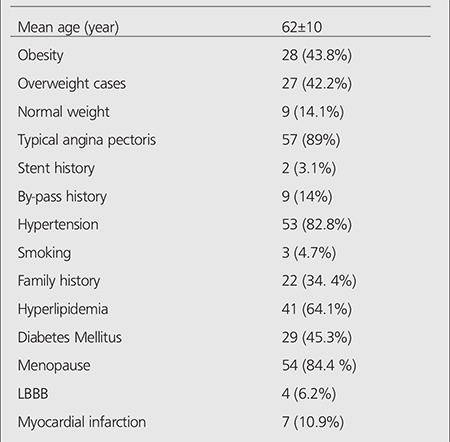
Cases’ demographic data (n=64)

**Table 2:**
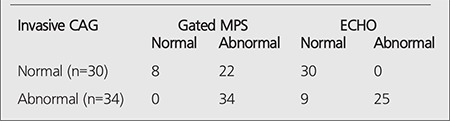
The number of patients of all 3 modalities: Gated MPS, ECHO and invasive CAG
